# Increasing cassava root yield: Additive-dominant genetic models for selection of parents and clones

**DOI:** 10.3389/fpls.2022.1071156

**Published:** 2022-12-16

**Authors:** Luciano Rogério Braatz de Andrade, Massaine Bandeira e Sousa, Marnin Wolfe, Jean-Luc Jannink, Marcos Deon Vilela de Resende, Camila Ferreira Azevedo, Eder Jorge de Oliveira

**Affiliations:** ^1^Department of Crop Science, Universidade Federal de Viçosa, Viçosa, Minas Gerais, Brazil; ^2^Embrapa Mandioca e Fruticultura, Cruz das Almas, Bahia, Brazil; ^3^Department of Crop, Soil and Environment Sciences, Auburn University, Auburn, AL, United States; ^4^Section on Plant Breeding and Genetics, School of Integrative Plant Sciences, Cornell University, Ithaca, NY, United States; ^5^United States Department of Agriculture – Agriculture Research Service, Plant, Soil and Nutrition Research, Ithaca, NY, United States; ^6^Department of Forestry Engineering, Universidade Federal de Viçosa, Viçosa, Minas Gerais, Brazil; ^7^Embrapa Florestas, Colombo, Paraná, Brazil; ^8^Department of Statistics, Universidade Federal de Viçosa, Viçosa, Minas Gerais, Brazil

**Keywords:** genomic selection, non-additive effects, dominance, breeding, breeding values, genotypic values

## Abstract

Genomic selection has been promising in situations where phenotypic assessments are expensive, laborious, and/or inefficient. This work evaluated the efficiency of genomic prediction methods combined with genetic models in clone and parent selection with the goal of increasing fresh root yield, dry root yield, as well as dry matter content in cassava roots. The bias and predictive ability of the combinations of prediction methods Genomic Best Linear Unbiased Prediction (G-BLUP), Bayes B, Bayes Cπ, and Reproducing Kernel Hilbert Spaces with additive and additive-dominant genetic models were estimated. Fresh and dry root yield exhibited predominantly dominant heritability, while dry matter content exhibited predominantly additive heritability. The combination of prediction methods and genetic models did not show significant differences in the predictive ability for dry matter content. On the other hand, the prediction methods with additive-dominant genetic models had significantly higher predictive ability than the additive genetic models for fresh and dry root yield, allowing higher genetic gains in clone selection. However, higher predictive ability for genotypic values did not result in differences in breeding value predictions between additive and additive-dominant genetic models. G-BLUP with the classical additive-dominant genetic model had the best predictive ability and bias estimates for fresh and dry root yield. For dry matter content, the highest predictive ability was obtained by G-BLUP with the additive genetic model. Dry matter content exhibited the highest heritability, predictive ability, and bias estimates compared with other traits. The prediction methods showed similar selection gains with approximately 67% of the phenotypic selection gain. By shortening the breeding cycle time by 40%, genomic selection may overcome phenotypic selection by 10%, 13%, and 18% for fresh root yield, dry root yield, and dry matter content, respectively, with a selection proportion of 15%. The most suitable genetic model for each trait allows for genomic selection optimization in cassava with high selection gains, thereby accelerating the release of new varieties.

## 1 Introduction

Cassava (*Manihot esculenta* Crantz) has great social and economic importance for Brazilian agriculture, where nearly 18.2 million tons were produced across 1.2 million hectares in 2020 (FAO, 2022). Most of the planted area is within small farms where the product is destined for on-farm consumption or local sales. However, with the starch price rising, there is a trend of increasing industry involvement in intensive cassava production. Although almost the entire plant can be used for human and animal consumption, farmers have chiefly focused on root production.

Cassava can be propagated by seeds or vegetatively by stem pieces (cuttings), with the former generally limited to breeding programs for allele recombination and generation of new hybrid combinations and the latter the most common method used by farmers for multiplication and root production (Ceballos et al., 2012). Once the F_1_ population is obtained, the hybrids are evaluated and selected regularly through several stages. Selection intensity and the evaluated traits depend on the amount of propagation material and the evaluation potential in different environments. According to Barandica et al. (2016), until the 21^st^ century, hybrid selection in early-phase breeding programs was performed visually without extensive phenotypic data collection. Therefore, until relatively recently, inheritance knowledge about relevant traits was very limited (Calle et al., 2005; Zacarias and Labuschagne, 2010; Ceballos et al., 2012; Tumuhimbise et al., 2014; Oliveira et al., 2015a).

In several phases of the breeding program, vegetative propagation allows the maintenance of high heterozygosity and phenotypic plasticity expression for several traits (Oliveira et al., 2015a). In addition, it allows hybrids to be evaluated and selected in different locations and crop seasons (Barandica et al., 2016), thus allowing the separation of genetic and environmental effects, through the effects of the genotype by environment interactions (Ceballos et al., 2016a; Bakare et al., 2022). Due to vegetative propagation and the high heterozygosity of the parents (Ceballos et al., 2016a), genetic variability within families represents approximately 90% of total genetic variability (Ceballos et al., 2016b), supporting the idea that elite clones can be obtained within any family.

One hypothesis that may explain this high intra-family variability is the presence of non-additive genetic effects, especially for yield traits (Calle et al., 2005; Jaramillo et al., 2005; Zacarias and Labuschagne, 2010; Parkes et al., 2013; Tumuhimbise et al., 2014). While the non-additive effects hamper clone and parent selection, they allow for exploration of heterosis, as the best hybrids can be multiplied by vegetative propagation and then be release as new varieties (Parkes et al., 2013). However, the low correlation between root yield performance in the initial and final stages of the breeding program prevents the early and accurate selection of the best hybrids in clonal evaluation trials (Barandica et al., 2016). As a result, large seedling populations are evaluated annually and selected for the next stages (Ceballos et al., 2012), with the goal of identifying the most promising genotypes in advanced phases of the breeding program. This greatly increases the costs of the variety development pipeline, as phenotypic measurements demand suitable infrastructure, skilled labor, and consequently large amounts of financial resources.

Progress in genotyping, especially in reducing costs and increasing marker density, is revolutionizing marker applications in plant breeding (Fergunson et al., 2012). Since Meuwissen et al. (2001), there have been high expectations of genomic selection implementation in multiple breeding programs, due to possible selection gain in situations where traditional evaluation methods are expensive, laborious, and/or inefficient (Crossa et al., 2013). In genomic selection, breeding populations are phenotyped and genotyped with high genomic coverage markers in order to allow prediction methods to predict genomic estimated breeding values (GEBVs) of each clone (Fergunson et al., 2012). According to Crossa et al. (2013), genomic selection can predict clones’ breeding values to accelerate recombination and their genotypic values as a means of targeting clones for advancement in the breeding pipeline.

For cassava, there is an expectation of genomic selection use for early selection in seedling trials as an alternative method to select traits that are difficult to measure or that demand high experimental accuracy (Oliveira et al., 2012), such as fresh root yield (FRY) and starch yield. In general, yield traits have predominantly non-additive effects (Jaramillo et al., 2005; Zacarias and Labuschagne, 2010; Parkes et al., 2013; Tumuhimbise et al., 2014) and low correlation of the phenotypic values obtained at initial phases (seedling and clonal evaluation trials) with those of advanced trials (uniform yield trials) (Barandica et al., 2016). Another trait of great importance in cassava is the dry matter content (DMC) in roots; its genetic heritability has predominantly been associated with additive effects (Jaramillo et al., 2005; Parkes et al., 2013; Tumuhimbise et al., 2014; Wolfe et al., 2016a), and high correlation between the different breeding program stages (Barandica et al., 2016). As a result, clone and parent selection in the seedling trials is less accurate for yield traits than for DMC. However, early selection for DMC may also increase breeding efficiency, even though phenotyping in seedling trials is time-consuming and laborious. This is because seedling trials involve the evaluation of thousands of clones, and there is limited root production per clone, which prevents the use of a simple method of evaluation (specific gravimetry).

When only the additive effects are considered in the parent selection, the progeny mean is equal to the mean of the parents’ breeding values; however, dominant effects prediction allows for heterosis exploration through parent complementarity (Almeida Filho et al., 2016). The genomic prediction of non-additive effects incorporated into genetic models increases the accuracy in parent and clone selection for low inheritance traits, as was observed in interspecific hybrid selection in *Eucalyptus* (Tan et al., 2018), intraspecific hybrids of *Pinus taeda* (Almeida Filho et al., 2016), maize (Lyra et al., 2019), inbred lines and crossbreed selection in Landrace and Yorkshire pigs (Esfandyari et al., 2016), and in clone selection of cassava (Wolfe et al., 2016a).

Genomic selection was also efficiently applied for predicting resistance to cassava mosaic disease, which displays a predominantly additive inheritance (Parkes et al., 2013; Tumuhimbise et al., 2014). In two years (annual breeding cycle), the allelic frequency of the marker with the greatest effect on cassava mosaic disease resistance rapidly increased from 44% to 66% (Wolfe et al., 2016b), much faster than the five or six years required in a conventional breeding cycle. Oliveira et al. (2012) noted that the two-year breeding cycle may have resulted in genetic gains higher than the conventional breeding cycle, of 56.9% and 39.92% for FRY and DMC, respectively.

Other important genomic selection goals are breeding population size reduction, time required to develop a new variety, and the ability to grow breeding populations outside the variety’s recommended location, allowing selection for biotic and abiotic disturbances outside the endemic region (Fergunson et al., 2012). New prediction methodologies are consistently being published (Meuwissen et al., 2001; Park and Casella, 2008; Habier et al., 2011; Legarra et al., 2011; Azevedo et al., 2015; Wolfe et al., 2021). Application of the appropriate methodology to a trait of interest may increase selection gains and simultaneously reduce the work required in phenotypic evaluations, which are mostly high in cost and low in yield (Fergunson et al., 2012). Wolfe et al. (2016a) have noted that a non-additive genomic relationship matrix may contribute to increased efficiency and yield in clone selection for traits with low heritability and/or that are difficult to measure.

Several studies have explored the efficiency of additive models of genomic selection. However, few have addressed the efficiency of dominant effects incorporated in genetic models for cassava breeding. Therefore, the objective of this work was to infer the efficiency of the G-BLUP, Bayes B, and RKHS genomic prediction methods with different genetic models for clone and parent selection to increase FRY, dry root yield (DRY) and DMC. Breeding program stages and genomic selection that may increase the efficiency of cassava breeding programs are also discussed.

## 2 Material and methods

### 2.1 Training population

The training population included 888 accessions belonging to the Cassava Germplasm Bank of Embrapa Cassava and Fruits (Cruz das Almas, Bahia, Brazil). This germplasm comprised 835 landraces and 53 improved varieties. One hundred and eighty accessions were characterized as sweet cassava (< 50 ppm of cyanogenic compounds), 136 as containing intermediary cyanide content (50–100 ppm cyanogenic compounds), 560 as bitter cassava (> 100 ppm cyanogenic compounds), and 12 as unclassified. These accessions were collected from all 26 Brazilian states, with every state represented by at least one genotype. The genotypes were evaluated in the cities of Cruz das Almas and Laje in the state of Bahia, Brazil, in 21 trials over a six-year period (2011 to 2016).

### 2.2 Phenotypic data collection

For most experiments, 15–20 cm stem cuttings were planted in double lines during the rainy season in the region (May–July). The experimental plot consisted of two rows of eight plants per row. The rows were 0.9 m apart, while plants in the same row were 0.8 m apart, with 11.52 m^2^ per plot. All recommended cassava cultural practices were employed (as in Souza et al., 2006). Trials were harvested 11–12 months after planting. The traits measured to estimate genomic selection efficiency were: 1) fresh root yield (FRY) at plot level (16 plants) and then adjusted to t.ha^-1^, 2) dry matter content in the roots (DMC), according to Kawano et al. (1987), where approximately 5 kg of roots were weighed in a hanging scale (WA) and then, the same sample was weighed with the roots submerged in water (WW). DMC was estimated utilizing the following formula: $DMC\left(\%\right)=\left(\frac{WA}{WA-WW}x158.3\right)-142$ and 3) dry root yield (DRY) in to t.ha^-1^, estimated per plot by multiplying the FRY and DMC.

A joint analysis of 21 trials with complete randomized block design or augmented block design were used to obtain the phenotypic data. Three replicates were used in the complete randomized block design, while in the augmented block design, 10–16 replicates of the common checks were used, with equal distribution of accession number per block. Improved clones (9602-02, 9607-07, 9824-09, 9655-02) and improved varieties (BRS Dourada, BRS Gema de Ovo, and BRS Novo Horizonte) were used as checks in different field trials. More details from the phenotypic dataset could be seen in **Table S1** and **S2**.

Due to unbalanced trials, we obtained the BLUP and deregressed BLUP (Garrick et al., 2009) for each clone. The BLUPs were obtained by the following mixed linear model: *y*_*ijl*_=*μ*+*c*_*i*_+*β*_*j*_+*r*_*l*(*j*)_+*ϵ*_*ijl*_ in which *y_ijl_* is the vector of phenotypic observations; *c_i_* is the clone random effect with $c_{i}\sim N\left(0,I{\hat{\sigma }}_{c}^{2}\right)$ *β_j_* is the combination of location and year, assumed as fixed effect; *r*_*l*(*j*)_ s the replication nested within location and year, assumed as random effect with $r_{j\left(l\right)}\sim N\left(0,I{\hat{\sigma }}_{r}^{2}\right)$ and ϵijl is the residual with $\epsilon_{ijl}\sim N\left(0,I{\hat{\sigma }}_{e}^{2}\right)$ The deregressed BLUPs were estimated by: $deregressed\;BLUP=\frac{BLUP}{1-\frac{PEV}{{\hat{\sigma }}_{c}^{2}}}$ Garrick et al., 2009), where the PEV is the prediction error variance of each clone and ${\hat{\sigma }}_{c}^{2}$ s the clonal variance component. The package lme4 (Bates et al., 2015) in R software version 3.5.2 (R Core Development Team, 2018) was used to obtain the BLUPs and deregressed BLUPs for each clone.

### 2.3 Genotyping and SNP quality control

DNA was extracted from cassava leaves following the CTAB (cetyltrimethylammonium bromide) protocol described by Doyle and Doyle (1987). To evaluate DNA integrity and standardize its concentration, 1.0% (w/v) agarose gels were stained with ethidium bromide (1.0 mg L^-1^) for visual comparison of a series of DNA phage Lambda (Invitrogen) concentrations. The DNA samples were sent to the Genomic Diversity Facility at Cornell University (http://www.biotech.cornell.edu/brc/genomic-diversity-facility) for genotyping-by-sequencing (GBS) (Hamblin and Rabbi, 2014). Genotypic data were selected using a minimum call rate of 0.90 and the missing markers were imputed by Beagle 4.1 software (Browning and Browning, 2016). Finally, SNPs with minor allele frequency (MAF) > 0.05 were retained. After applying marker quality control, 48,655 SNPs were selected for genomic prediction.

### 2.4 Genomic selection methods and genetic models

The genomic best linear unbiased prediction (G-BLUP), Reproducing Kernel Hilbert Spaces (RKHS), and Bayes B prediction methods were evaluated, considering the additive (A) and additive-dominant (A+D) genetic models, except RKHS, which predicts genetic effects based on non-parametric—and thus neither additive nor dominance—covariances. The additive-dominant genetic model of G-BLUP is expressed: *y*_*d*_=*Jμ*+*Za*+*Hd*+*ϵ* where yd is the deregressed BLUP vector; µ is the general mean; a is the additive effect vector, random $a\sim N\left(0,G{\hat{\sigma }}_{a}^{2}\right)$ d is the dominant deviation effect vector, random $d\sim N\left(0,D{\hat{\sigma }}_{d}^{2}\right)$ ϵ is the residual effect vector, $\epsilon \sim N\left(0,I{\hat{\sigma }}_{e}^{2}\right)$ J, Z and H are the incidence matrices for µ, a and d, respectively, as *COV*(*a*,*d*)=0 The additive relationship matrix G was: $G=\frac{ZZ'}{2\sum^{}p_{i}\left(1-p_{i}\right)}$ in which *Z* is the marker matrix (-1, 0 and 1) and *p_i_* is the major allele frequency of *i* marker. Two additive-dominant genetic models were tested for the G-BLUP method, the Classical (Vitezica et al., 2013) and the Genotypic (Su et al., 2012), differing in the parameterization of the genomic relationship matrix due to dominance. The Classical dominant relationship matrix was parameterized by the following Vitezica et al. (2013):


$$H=\{\begin{array}{c} if\;MM:-q^{2}\\ if\;Mm:2pq\\ if\;mm:-p^{2} \end{array}$$

$$D=\frac{HH'}{2\sum^{}p_{i}q_{i}\left(1-p_{i}q_{i}\right)}.$$

The Genotypic dominant relationship matrix was estimated by the following equation (Su et al., 2012):


$$H^{*}=\{\begin{array}{c} if\;MM:-2pq\\ if\;Mm:p^{2}+q^{2},\\ if\;mm:-2pq \end{array}$$

D*=H*H*'2∑​piqi(1−piqi),

For the Bayes B method, the complete conditional prior distribution was used: $y_{d_{i}}|a_{j},d_{j},Z_{i\times j},H_{i\times j}\sim N\left(\mu +\sum_{j}Z_{i\times j}a_{j}+\sum_{j}H_{i\times j}d_{j},{\hat{\sigma }}_{e}^{2}\right)$ in which yd is the deregressed BLUP vector; µ is the general mean; *a*_*j*_ nd *d*_*j*_ re the additive and dominant marker effects, both random $a_{j}|{\hat{\sigma }}_{a_{j}}^{2}\sim N\left(0,I{\hat{\sigma }}_{a_{j}}^{2}\right)$ $d_{j}|{\hat{\sigma }}_{d_{j}}^{2}\sim N\left(0,I{\hat{\sigma }}_{d_{j}}^{2}\right)$ and *COV*(*a*_*i*_,*d*_*i*_)=0 Z and H are the incidence matrix of *a*_*j*_ and *d*_*j*_ respectively.

The model of the RKHS method was: *y*_*d*_=*Jμ*+*Xg*+*ϵ* where yd is the deregressed BLUP vector; µ is the general mean; g is the genotypic effect vector, random $g\sim N\left(0,K{\hat{\sigma }}_{g}^{2}\right)$ ϵ is the residual effect vector, $\epsilon \sim N\left(0,I{\hat{\sigma }}_{e}^{2}\right)$ J and X are the incidence matrix of µ and g, respectively. K is a gaussian matrix estimated by: $K=exp\left(\frac{-hD}{median\left(D\right)}\right)$ h is the reduction coefficient to K values (in this work h was equal to 1), and D is the Euclidian distance of *Z* codified marker matrix (Gianola et al., 2006; Crossa et al., 2010).

The 5-fold cross-validation with three repetitions was performed to estimate the following parameters: 1) predictive ability $\left({\hat{r}}_{\hat{y}y}=\hat{COR}\left({\hat{Pred}}_{Val},BLUP_{Val}\right)\right)$ in which ${\hat{Pred}}_{Val}$ are the genomic estimated breeding values (GEBVs) for additive genetic models, or genomic estimated genotypic values (GEGVs) for additive-dominant and RKHS models, and *BLUP*_*Val*_ are the BLUPs from the validation population; 2) bias $\left(\hat{b}=\hat{COV}\left({\hat{Pred}}_{Train},BLUP_{Train}\right)/{\hat{\sigma }}_{Pred_{Train}}^{2}\right)$ in which ${\hat{Pred}}_{Train}$ are the genomic estimated breeding values (GEBVs) for additive genetic models, or genomic estimated genotypic values (GEGVs) for additive-dominant genetic models, of the training population, *BLUP*_*Train*_ are the BLUPs from the training population, ${\hat{\sigma }}_{Pred_{Train}}^{2}$ s the variance of the GEBVs for additive genetic models, or genomic estimated genotypic values (GEGVs) for additive-dominant genetic models of the training population; 3) broad-sense genomic heritability $\left({\hat{H}}^{2}={\hat{\sigma }}_{g}^{2}/\left({\hat{\sigma }}_{g}^{2}+{\hat{\sigma }}_{e}^{2}\right)\right)$ in which ${\hat{\sigma }}_{g}^{2}$ s the genomic variance, ${\hat{\sigma }}_{e}^{2}$ s the residual variance; 4) narrow-sense genomic heritability $\left({\hat{h}}^{2}={\hat{\sigma }}_{a}^{2}/\left({\hat{\sigma }}_{g}^{2}+{\hat{\sigma }}_{e}^{2}\right)\right)$ which ${\hat{\sigma }}_{a}^{2}$ s the additive genomic variance, ${\hat{\sigma }}_{g}^{2}$ s the genomic variance, ${\hat{\sigma }}_{e}^{2}$ s the residual variance. For each replicate of the cross-validation process, the population was split into five equal folds. Five genomic predictions were performed per fold used as test set (no phenotypes) each fold was predicted by the remaining four-folds training set (with phenotypes).

The *sommer* R package (Covarrubias-Pazaran, 2016) was used to fit the G-BLUP and RKHS models, while the *BGLR* R package (Perez and De Los Campos, 2014) was used to fit the Bayes B model. All methods were performed using R software version 3.5.2 (R Core Development, 2018). For Bayes B method, we ran 20,000 Markov Chain Monte Carlo (MCMC) iterations with the burn-in of the initial 4,000 iterations and thinning of 10, we applied different priori for π for each trait and genetic model, these values were previously estimated by Bayes Cπ (Table S3).

The training-validation partitions of the population used in cross-validation were set up to be identical across prediction models, using the set.seed() function of R software version 3.5.2 (R Core Development, 2018). The residual variances of Markov Chain Monte Carlo (MCMC) of the Bayes B method were used to evaluated the MCMC convergency by the Raftery and Lewis’s convergence diagnostic (Raftery and Lewis, 1992) applied in coda R package (Plummer et al., 2006).

### 2.5 Analysis of variance and Tukey’s multiple comparison test

Analysis of variance was performed to estimate the effects of the genomic selection methods for predictive ability and bias estimates for DMC, FRY, and DRY. These analyses were performed using the *lme4* R package (Bates et al., 2015).

The following mixed model was used to estimate the efficiency of the genomic selection methods: *y*_*ijk*_=*m*_*i*_+*s*_*jk*_+*e*_*ijk*_ which y is the dependent variable, as predictive ability and bias; mi is the mean of the genomic selection method I, assumed as fixed effect; s_jk_ is the effect of cross validation of the replication j and fold k, assumed as random effect $s\sim \left(0,{\hat{\sigma }}_{cv}^{2}\right)$ and eijk is the residual effect of the i genomic selection method of the j replication and k fold, $e\sim \left(0,{\hat{\sigma }}_{e}^{2}\right)$ The genomic prediction means were submitted to the Tukey multiple comparison test implemented in the *emmeans* R package (Russel, 2018).

### 2.6 Cohen’s Kappa coefficient

The Cohen’s Kappa coefficient (Cohen, 1960) was used to analyze the coincidence of clone selection by the different genomic selection methods, considering a selection proportion (SP) amplitude ranging from 5–30%. The coincidence selection was performed using a binary code and the selected and unselected individuals received code “1” and “0”, respectively. The Kappa coefficient and coincidences selection index were calculated using R.

## 3 Results

### 3.1 Efficiency of the genomic selection methods and genetic models

In general, the inclusion of the dominant genetic effects increased the genomic variance explained by the markers (**Table 1**), and reduced the genomic additive variance and residuals (**Table 1** and **Figure S1**). Smaller changes in the broad-sense genomic heritability were observed for DMC, except for the Bayes B method, which demonstrated the highest broad-sense genomic heritability among the prediction methods with an additive-dominant genetic model.

**Table 1:** Means of the genetic parameters estimated by different genomic prediction methods for fresh root yield (FRY), dry root yield (DRY), and dry matter content (DMC) in roots of cassava.

Traits / Prediction methods	Genetic parameters					
Fresh root yield	*ĥ^2^*	*Ĥ^2^*	${\hat{\sigma }}_{a}^{2}$	${\hat{\sigma }}_{g}^{2}$	${\hat{\sigma }}_{d}^{2}$	${\hat{\sigma }}_{e}^{2}$
G-BLUP A^1^	0.347	–	17.0	–	17.0	32.0
G-BLUP A+D Classical^2^	0.139	0.386	6.4	11.5	17.9	28.5
G-BLUP A+D Genotypic^3^	0.053	0.400	2.6	16.8	19.4	29.1
RKHS	–	0.520	–	–	31.6	29.0
Bayes B A^4^	0.582	–	43.9	–	43.9	31.2
Bayes B A+D^5^	0.257	0.734	26.2	49.2	75.4	26.9
Dry root yield	*ĥ^2^*	*Ĥ^2^*	${\hat{\sigma }}_{a}^{2}$	${\hat{\sigma }}_{g}^{2}$	${\hat{\sigma }}_{d}^{2}$	${\hat{\sigma }}_{e}^{2}$
G-BLUP A^1^	0.332	–	1.39	–	1.39	2.81
G-BLUP A+D Classsical^2^	0.175	0.369	0.71	0.79	1.49	2.55
G-BLUP A+D Genotypic^3^	0.096	0.381	0.40	1.20	1.60	2.59
RKHS	–	0.504	–	–	2.61	2.57
Bayes B A^4^	0.571	–	3.69	–	3.69	2.74
Bayes B A+D^5^	0.262	0.728	2.32	4.15	6.46	2.39
Dry matter content	*ĥ^2^*	*Ĥ^2^*	${\hat{\sigma }}_{a}^{2}$	${\hat{\sigma }}_{g}^{2}$	${\hat{\sigma }}_{d}^{2}$	${\hat{\sigma }}_{e}^{2}$
G-BLUP A^1^	0.517	–	2.10	–	2.10	
G-BLUP A+D Classical^2^	0.477	0.522	1.92	0.18	2.10	1.92
G-BLUP A+D Genotypic^3^	0.457	0.525	1.86	0.27	2.13	1.92
RKHS	–	0.504	–	–	2.61	2.57
Bayes B A^4^	0.673	–	4.04	–	4.04	1.95
Bayes B A+D^5^	0.325	0.792	2.73	3.99	6.72	1.75

${\hat{h}}^{2}$ , narrow-sense genomic heritability; ${\hat{H}}^{2}$ , broad-sense genomic heritability; ${\hat{\sigma }}_{a}^{2}$ , additive genomic variance; ${\hat{\sigma }}_{d}^{2}$ , dominant genomic variance; ${\hat{\sigma }}_{g}^{2}$ , genomic variance; ${\hat{\sigma }}_{e}^{2}$ , residual variance. ^1^G-BLUP with additive model; ^2^G-BLUP with additive-dominant model, classical dominant relationship matrix (Vitezica et al., 2013); ^3^G-BLUP with additive-dominant model, genotypic dominant relationship matrix (Su et al., 2012); ^4^Bayes B with additive model; ^5^Bayes B with additive-dominant model.

Insert **Table 1**

A predominance of additive effects for DMC was identified with the G-BLUP method (**Table 1** and **Figure S1**), while for FRY and DRY the dominant effects prevail. The Bayes B method showed the highest estimates of broad-sense genomic heritability and genomic variance components. However, the variation of the broad-sense genomic heritabilities between traits was smaller, suggesting a relatively large proportion of dominance variance. Even with the highest broad-sense genomic heritability, the Bayes B A+D method exhibited smaller narrow-sense genomic heritability than the G-BLUP A+D method, regardless of the dominant relationship matrix used (**Table 1**). However, all the additive-dominant genetic models overestimated the broad-sense genomic heritability because it was higher than the phenotypic heritability (0.337, 0.351, and 0.545 for FRY, DRY, and DMC, respectively).

**Figure 1:**
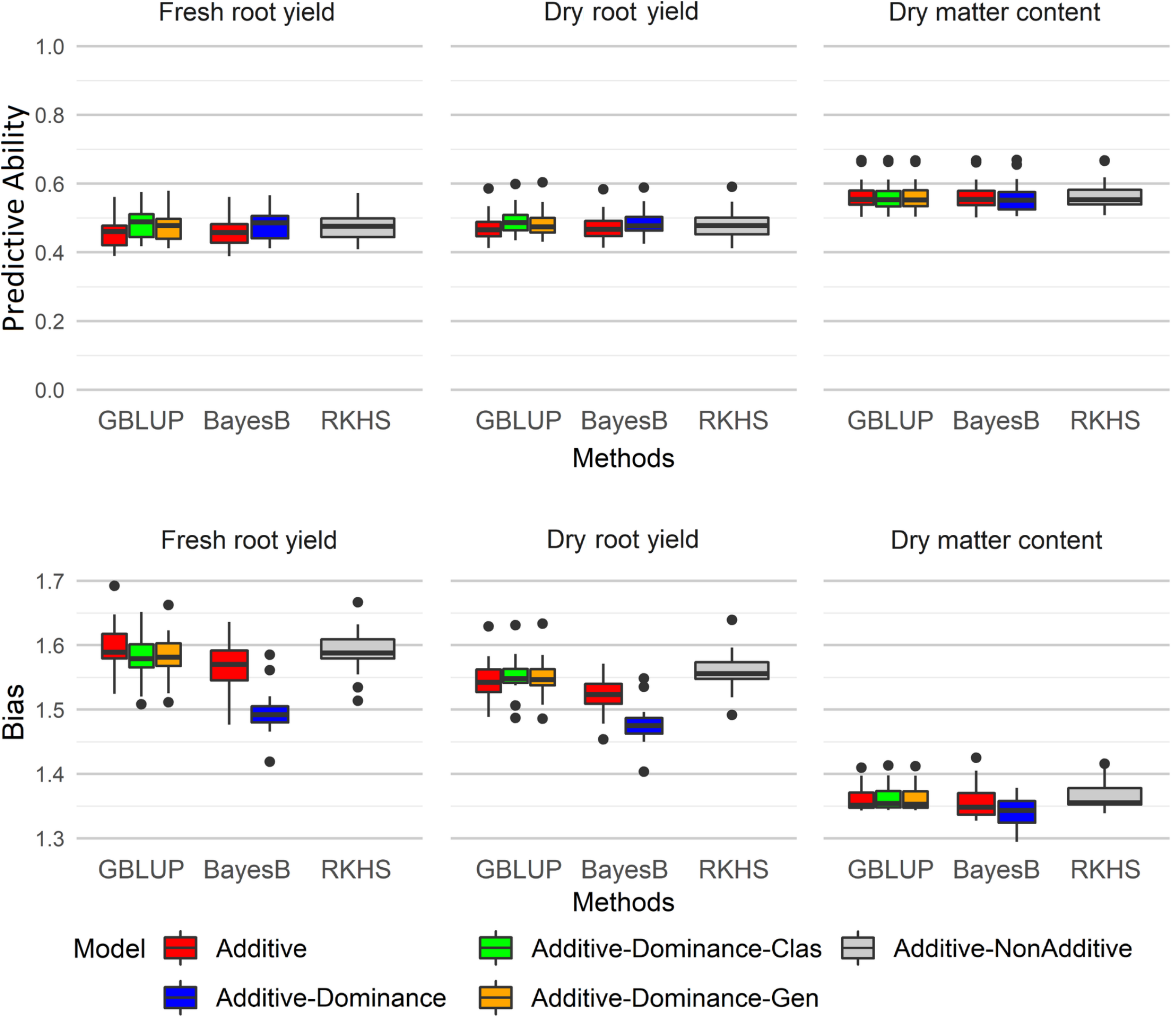
SP: selection proportion; SD GS: genomic selection differential; SD PS: phenotypic selection differential; GB/PB: ratio between the breeding cycle assisted by genomic selection and conventional breeding cycle; Efficiency = *SD* *GS*/[*SD* *PS*×(*GB*/*PB*)] Boxplots of predictive ability and bias for different genomic selection methods (G-BLUP, Bayes B, and RKHS) with additive and additive-dominant genetic models for fresh root yield (FRY), dry root yield (DRY), and dry matter content (DMC). GBLUP, genomic best linear unbiased prediction; RKHS, reproducing kernel Hilbert spaces.

The additive-dominant genetic models showed higher predictive ability than additive models and RKHS method for yield traits (FRY and DRY, **Figure 1**). The highest predictive ability was demonstrated by the G-BLUP A+D classical method (average of 0.484 for FRY and 0.492 for DRY), followed by Bayes B A+D (average of 0.479 for FRY and 0.488 for DRY). In addition, the predictions of dominant effects in genetic models for yield traits reduced the bias estimate, with the smaller bias at Bayes B method (**Figure 1**). The RKHS method showed the highest bias estimates for all traits.

### 3.2 Analysis of variance and Tukey’s multiple comparison test of the different genomic selection methods

Significant differences between the genomic selection methods with different genetic models were identified for predictive ability and bias for all agronomic traits except the predictive ability of DMC (**Table 2**). Although there were no significant differences in the predictive ability between the genomic selection methods with additive-dominant models, the G-BLUP A+D classical method showed the highest predictive ability for FRY (0.483) and DRY (0.492) (**Table 2**). Bayes B A+D and RKHS methods did not show significant differences for predictive ability in comparison with G-BLUP A+D classical method for DRY. On the other hand, for FRY only Bayes B A+D and G-BLUP A+D genotypic methods did not show significant differences with the G-BLUP A+D classical method.

**Table 2:** Analysis of variance (ANOVA) and Tukey’s multiple comparison test (p ≤ 0.05) for prediction parameters of different genomic selection methods for fresh root yield (FRY), dry root yield (DRY), and dry matter content (DMC) in cassava.

ANOVA	DF	Fresh root yield			Dry root yield			Dry matter content	
		${\hat{r}}_{\hat{y}y}$	$\hat{b}$		${\hat{r}}_{\hat{y}y}$	$\hat{b}$		${\hat{r}}_{\hat{y}y}$	$\hat{b}$
Methods	5	21.91*	51.76*		10.95*	50.35*		2.07	9.28*
Tukey multiple comparison test									
Bayes B A		0.458C	1.568B		0.474C	1.522B		0.566A	1.357B
Bayes B A+D		0.479AB	1.497A		0.488AB	1.477A		0.561A	1.340A
G-BLUP A		0.457C	1.598C		0.474C	1.547C		0.567A	1.360B
G-BLUP A+D Classical		0.483A	1.580BC		0.492A	1.552C		0.564A	1.362B
G-BLUP A+D Genotypic		0.474B	1.582BC		0.485AB	1.550C		0.565A	1.361B
RKHS		0.476AB	1.590C		0.482BC	1.560C		0.567A	1.366B

${\hat{r}}_{\hat{y}y}$ , predictive ability; $\hat{b}$ , bias, DF, degrees of freedom. *significant by chi-square test (p ≤ 0.05). Upper case letters means significant difference between genomic selection ethods for the Tukey multiple comparison test (p ≤ 0.05) : predictive ability; : bias, DF: degrees of freedom. *significant by chi-square test (p≤0.05). Upper case letters means significant difference between genomic selection methods for the Tukey multiple comparison test (p≤0.05).

Among the methods with non-additive effects, the G-BLUP A+D classical was significantly different from the RKHS method for DRY but not for FRY. As the RKHS method can predict additive and partial epistatic effects (Gianola et al., 2006; Crossa et al., 2010), it is possible that the epistatic effects were more important for FRY than DRY, as the RKHS method did not show a significant difference with the additive genetic models G-BLUP A and Bayes B A (**Table 2**).

DMC showed the highest phenotypic heritability and predictive ability of traits. However, there was no improvement in predictive ability when the additive-dominant genetic models were used to predict this trait, which reinforced the theory that DMC in cassava has a high influence from additive effects. On the other hand, for FRY and DRY, the additive-dominant models demonstrated increased predictive ability, suggesting a greater importance of dominant effects for these traits in cassava.

### 3.3 Expected genetic gains from different genomic prediction methods through different selection proportion

**Figure 2:**
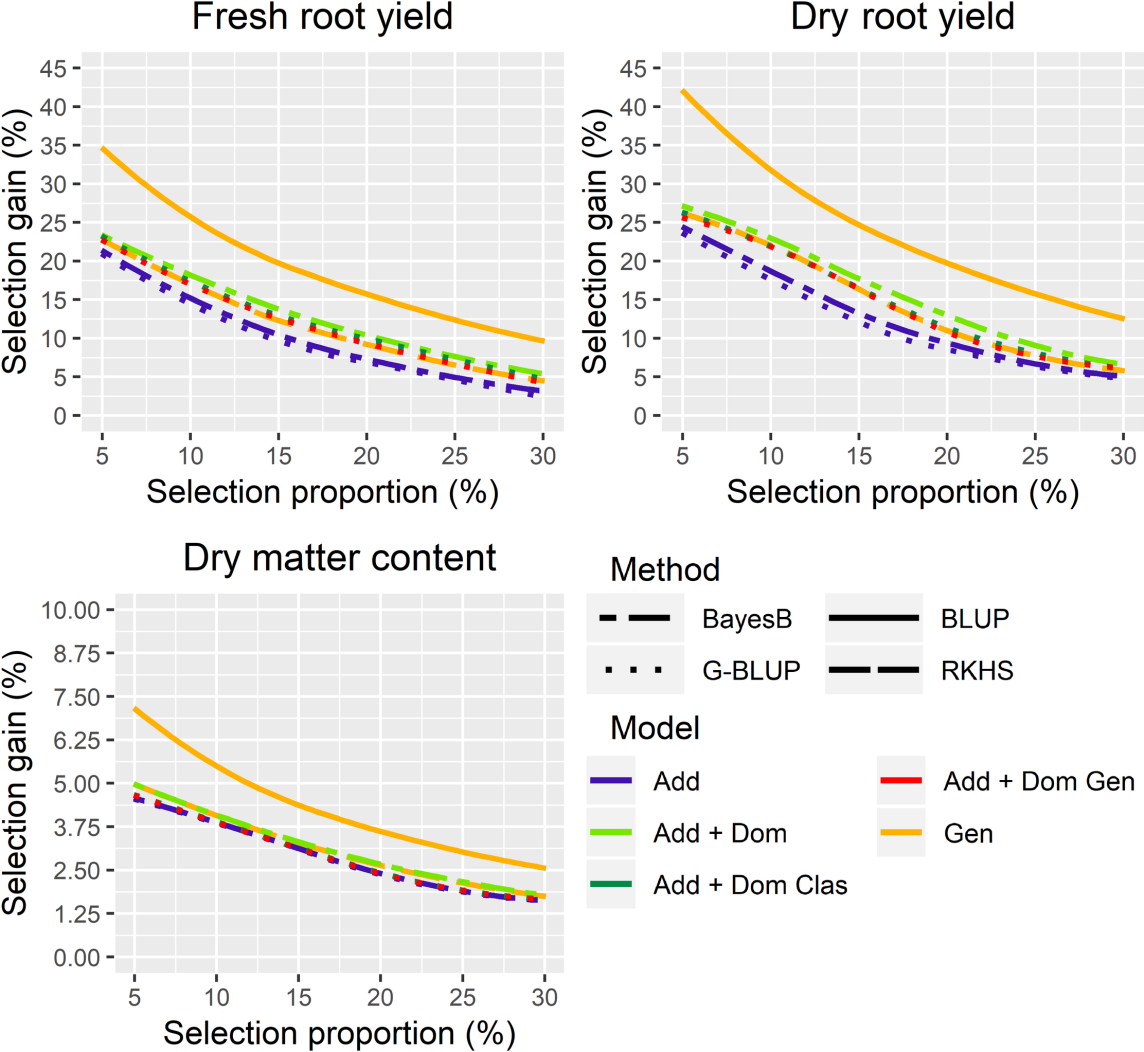
Expected selection gains for combinations of different genomic prediction methods and genetic models for fresh (FRY) and dry root yield (DRY) and dry matter content (DMC) in the roots of cassava, considering a selection proportion ranging from 5 to 30%. G-BLUP: genomic best linear unbiased prediction method; BLUP: phenotypic best linear unbiased prediction method; RKHS: reproducing kernel Hilbert spaces method; Add: additive; Add + Dom, Additive and dominant genetic model; Add + Dom Clas, Additive and dominant classical genetic model; Add + Dom Gen, Additive and dominant genotypic genetic model; Gen, genotypic model.

Although significant differences were detected between genomic prediction methods with different genetic models by ANOVA and Tukey’s mean test (**Table 2**), the expected genetic gains for genomic prediction were still smaller than those obtained by phenotypic selection, with expected selection gains equivalent to 67.5%, 67.1%, and 69.4% of the phenotypic selection for FRY, DRY, and DMC, respectively (**Figure 2**). Although selection gains with genomic predictions were similar for all traits, the non-additive genetic models, such as Bayes B A+D, RKHS, and G-BLUP A+D classical and genotypic, increased the gain by an average of 0.69 t/ha for FRY and 0.24 t/ha for DRY in comparison with the additive genetic models. For DMC, the differences between the selection gains of genomic prediction methods were lower (average of 0.04%), because there was no significant difference between the clone prediction methods for this trait (**Table 2**). Moreover, the selection differential for DMC in the roots was lower than for yield traits due to the smaller trait amplitude (17–38%).

There was a great uniformity in the differences between the selection gains of the phenotypic BLUP and the predicted gains in the different selection proportions, with a mean difference of selection gain of 6.18% and 7.79% of the Bayes B A+D model for FRY and DRY, respectively (**Figure 2** and **Table S2**). For DMC, there were lower gains differences between the phenotypic selection and genomic prediction, with the largest difference observed in the G-BLUP A method (average of 1.40% of genetic gain) compared to others (**Figure 2** and **Table S4**).

The genomic expected selection gain and its relative efficiency to phenotypic expected selection gain were calculated. According to Oliveira et al. (2012), the conventional breeding cycle of cassava is at least four years due to the need to include phenotypic information from a minimum of four breeding phases (clonal evaluation trial, preliminary yield trial, advanced yield trial, and uniform yield trial). The efficiency and the selection gains per time unit to simulate early selection assisted by genomic selection were calculated. The efficiency was determined by comparing time required to recombine the selected clones as parents in a conventional breeding program vs. one assisted by genomic selection.

Genomic selection based on the G-BLUP A+D classical method for FRY and G-BLUP A for DMC was more efficient than phenotypic selection when the breeding cycle was ≤ 0.60 of the conventional breeding cycle (**Table 3** and **Figure S2**). However, for DRY the genomic selection was more efficient than phenotypic selection only with a selection proportion of 5–15%.

**Table 3:** Relative efficiency of genomic selection compared to phenotypic selection using different selection proportions with the G-BLUP A+D classical method for fresh root yield (FRY), dry root yield (DRY), and dry matter content (DMC) in cassava.

	Fresh root yield						Dry root yield						Dry matter content					
Method	G-BLUP Additive Dominant Classical						G-BLUP Additive Dominant Classical						G-BLUP Additive					
SP (%)	5%	10%	15%	20%	25%	30%	5%	10%	15%	20%	25%	30%	5%	10%	15%	20%	25%	30%
SD GS	23.8	17.2	13.0	9.9	7.4	4.7	25.8	22.0	16.7	11.3	8.4	5.8	4.6	3.9	3.1	2.4	1.9	1.6
SD PS	35.1	25.4	19.8	15.7	12.4	9.6	42.8	31.7	24.7	19.7	15.8	12.5	7.3	5.5	4.4	3.6	3.0	2.5
GB/PB	Efficiency						Efficiency						Efficiency					
1.00	0.68	0.68	0.66	0.63	0.60	0.49	0.60	0.69	0.68	0.57	0.53	0.47	0.64	0.71	0.71	0.67	0.62	0.62
0.80	0.85	0.84	0.82	0.79	0.75	0.61	0.75	0.87	0.84	0.72	0.66	0.59	0.79	0.88	0.89	0.83	0.78	0.78
0.65	1.04	1.04	1.01	0.97	0.92	0.75	0.93	1.07	1.04	0.88	0.82	0.72	0.98	1.09	1.09	1.02	0.96	0.96
0.60	1.13	1.13	1.10	1.06	1.00	0.81	1.00	1.16	1.13	0.95	0.88	0.78	1.06	1.18	1.18	1.11	1.04	1.04
0.40	1.70	1.69	1.65	1.58	1.50	1.22	1.51	1.74	1.69	1.43	1.33	1.17	1.59	1.77	1.77	1.66	1.56	1.56
0.20	3.40	2.70	2.63	2.53	2.40	1.95	2.41	2.78	2.70	2.29	2.12	1.87	2.54	2.83	2.83	2.66	2.49	2.49

SP, selection proportion; SD GS, genomic selection differential; SD PS, phenotypic selection differential; GB/PB, ratio between the breeding cycle assisted by genomic selection and conventional breeding cycle; Efficiency = SD GS/[*SD PS*×(GB⁄PB)].

Reducing breeding cycle time by 60% using genomic selection could result in gains of 65%, 69%, and 77% over those provided by phenotypic selection for FRY, DRY, and DMC, respectively, in a selection proportion of approximately 15% of the best clones (**Table 3**). If the breeding cycle was reduced to 20% of the conventional breeding cycle (four years to ten months), the genetic gains would be 163%, 170%, and 183% over those provided by phenotypic selection for FRY, DRY, and DMC, respectively.

The selection proportion affected significantly the relative efficient of the genomic prediction only in breeding cycle time reductions biggest then 35% of the conventional Cassava breeding cycle (**Table 3**).

## 4 Discussion

### 4.1 Phenotypic and genomic heritability and its implications for genomic selection

According to Oliveira et al. (2015b), heritability estimates can assist selection strategies in increasing genetic gain, as well as defining the breeding method and experimental design. Given the broad- and narrow-sense genomic heritability, the G-BLUP A+D classical method showed that cassava yield traits demonstrate a predominance of dominant effects. In addition, the broad-sense genomic heritability of G-BLUP A+D was closer to the phenotypic heritability values (0.337 for FRY, 0.351 for DRY, and 0.545 for DMC [**Table 1**]). Stability of FRY and DRY are important agronomic attributes for any cassava variety to ensure high market competitiveness in the starch industry, especially as there is a minimum acceptable DMC threshold for processing the raw material. Roots with DMC index below this threshold are not processed by the starch industry due to the high industrial cost and low starch yield.

Knowledge about trait heritability and variation gained during field evaluation in different environments may assist in optimizing selection of cassava breeding programs, with the goal of developing new cassava varieties with higher starch yield stability. Optimizing the selection proportion and evaluated traits in each breeding phase can maximize the probability of selecting the best clone. This is because low heritability traits such as FRY and starch yield are generally evaluated in the final breeding phases due to greater stem cutting availability (more plants per plot across multiple locations).

Wolfe et al. (2016b) also related the predominance of additive and dominant deviation effects for DRY and FRY, respectively. They found similar estimates of broad- and narrow-sense heritability for the first genomic selection cycle of IITA population using the G-BLUP A+D method (0.12 and 0.35 for narrow- and broad-sense heritability, respectively, for FRY, and 0.47 and 0.52 for narrow and broad-sense heritability, respectively, for DMC). Wolfe et al. (2016b) found that the appropriate genetic model for DMC was the additive-dominant, while in the present study the additive-dominant models obtained similar results to the other models’. The reduction of the variance explained by the additive component was noted previously in cassava (Wolfe et al., 2016b) and other species such as *Pinus taeda* L. (Muñoz et al., 2014) and hybrids of *Eucalyptus urophylla* and *E. grandis* (Bouvet et al., 2016). Several authors reported that during prediction using additive genetic models, part of the dominant deviation was predicted along with the additive effects; however, when using additive-dominant genetic models, this dominant deviation predicted by the additive effects is then computed by the dominant variance (Zuk et al., 2012; Vitezica et al., 2013; Muñoz et al., 2014; Wolfe et al., 2016a). According to Vitezica et al. (2013) genetic models with assumptions of additive and dominant deviation effects result in better genomic predictions.

### 4.2 Efficiency of cassava selection considering different genomic prediction methods and genetic models

There were significant differences in predictive ability between the methods for FRY and DRY, mainly due to different genetic models (additive and non-additive). The additive-dominant genetic models showed higher predictive ability than additive genetic models for FRY and DRY. Among the genomic selection methods, the G-BLUP A+D classical (Vitezica et al., 2013) had high predictive ability and low bias, statistically similar to other additive-dominant genetic models. Therefore, the additive-dominant genetic models allow for exploration of part of the non-additive effects by increasing cassava clone selection accuracy. Other authors have evaluated relationship matrices with classical and genotypic dominant models and verified the lack of differences in the genomic predictions of these matrices, although the broad-sense heritability has been somewhat lower in the matrix (H*) of genotypic dominant (Vitezica et al., 2013; Wolfe et al., 2016a). However, the correlation between the additive and dominant parameters was higher in the G-BLUP genotypic method in comparison with the classical one (Wolfe et al., 2016a), which corroborates the correlations found in this work.

Dominance effects occurs due to the interaction between alleles at the same locus and its main benefits are expected in crossbreeding, since dominance has been suggested as one of the genetic mechanisms explaining heterosis (Shull, 1908). Indeed, hybrid vigor for yield components in cassava over better-parent values has been reported (Parkes, et al., 2020), indicating that heterosis should be explored in order to develop superior cassava genotypes. Therefore, genomic predictions for traits such as FRY and DRY must be based on the assumption that non-additive effects are an important component that should be considered in the predictions to optimize crossing designs, such as in mate-pair allocation (Almeida Filho et al., 2019). As a further step, the role of dominance effects on the genetic architecture of FRY and DRY should be evaluated in the breeding population generated in this study.

For DMC, there was no significant difference between genetic models for predictive ability, although the additive genetic models showed better prediction abilities than additive-dominant models. Similar results were reported in the first genomic selection cycle of IITA cassava population (Wolfe et al., 2016a). According to Denis and Bouvet (2013) the decision of which genetic model to use in genomic selection depends on the training population as well the traits under selection. Specifically, in cassava, the genomic prediction of FRY and DRY would be more efficient if applying additive-dominant genetic models, while for DMC the additive models are satisfactory. Among genetic models, additive gene action had highest response to selection. Therefore, in the case of DMC, the population improvement focused on genetic additive effects can achieve large medium‐to‐long term genetic gains.

Incorporating non-additive effects into the genetic model reduces the additive genomic variance and the bias of the GEBVs, as well increasing the accuracy for selecting the best parent (Vitezica et al., 2013; Wolfe et al., 2016a). On the other hand, some simulated studies did not find any differences in predictive ability of GEBVs between additive and additive-dominant genetic models (Almeida Filho et al., 2016; Heidaritabar et al., 2016). Therefore, it is expected that non-additive effects prediction may increase the genetic gains for yield traits of new cassava varieties in the breeding programs (Muñoz et al., 2014; Wolfe et al., 2016a).

The expected selection gains for FRY and DRY were high due to the training population being composed of germplasm accessions with high genetic variability (**Figure S2**) for several traits, including yield traits (Oliveira et al., 2015a; Oliveira et al., 2016). Within a group of clones that deviated from the FRY and DRY mean (**Figure S2**), some were from high-yield, improved varieties (FRY potential of > 30 t/ha) such as BRS Novo Horizonte, BRS Poti Branca, BRS Kiriris, and BRS Tapioqueira.

According to the Bayes B A+D method, if the cassava breeding cycle was reduced by 40%, the genomic selection gains would be on average 12.48% and 11.92% higher than phenotypic selection gains for FRY and DRY, respectively. A similar observation was made for DMC (22.16%). Oliveira et al. (2012) reported that with a 25% reduction in breeding cycle time, the relative efficiency of RR-BLUP genomic prediction was 4.6%, 15.96%, and -7.05% for FRY, starch yield, and DMC, respectively. According to these authors, higher selection gains may be achieved by reducing the time required to identify and recombine the parents in the breeding cycle. These results may assist in the planning of genomic selection implementation to increase the frequency of new cassava varieties with good agronomic traits and adaptations to new biotic and abiotic stress challenges.

Reducing the selection proportion is not feasible as reducing breeding cycle time to improve genetic gain, but it may be the next milestone to improve genetic gain in cassava breeding, by increasing the number of clones evaluated in earlier stages by genomic prediction.

### 4.3 Potential application of genomic selection in cassava breeding

A previously recommended method for clone and parent selection in seedling trial phases was assessment of the harvest index (the ratio of FRY and the biomass yield), used for FRY indirect selection in seedling nursery trials and clonal evaluation trials (Kawano et al., 1998) due to its high correlation (0.730). However, when analyzing the historical data (2000–2013) of the cassava breeding program at the International Center for Tropical Agriculture (CIAT), Barandica et al. (2016) found very low correlation between FRY and harvest index (0.11). In addition, the harvest index assessment is more labor-intensive than the FRY evaluation alone. According to Barandica et al. (2016) the correlation of FRY between the clonal evaluation trials and the uniform yield trials was 0.29, while in the present study the correlation between the GEBVs and the uniform yield trials for the G-BLUP A+D classical method was 0.483. In future studies, the efficiency of genomic selection in the seedling trial phase for FRY could be better understood by determining realized genetic gains.

Ceballos et al. (2016a) stated that one issue in the selection of good parents is the high intra-family genetic variability due to high heterozygosity. Thus, new cassava varieties may derive from crosses between parents with low agronomic performance. Indeed, Kawano et al. (1998) evaluated almost 327,000 clones from 4,130 crosses during 14 years of research, and among all those evaluated clones only three were officially released as new varieties.

Commonly the standard methods used for parent selection are the *per se* performance and, less commonly, general combining ability (Ceballos et al., 2004). Unfortunately, there is no linear relationship between the *per se* performance and the progeny’s breeding values due to dominant deviation (Ceballos et al., 2004). In addition, the diallel analysis in cassava breeding programs is problematic because the crosses in cassava are laborious and usually imbalanced due to issues of flowering synchronization (Ceballos et al., 2017), the considerable unpredictability of the flowering season (between four to ten months after planting), and the time for seed maturity after harvest demanding at least one year to obtain the seeds of controlled crossings (Ceballos et al., 2004). Su et al. (2012) reported that genetic models with additive and non-additive effects prediction might allow for exploitation of specific combining ability. Therefore, applying genomic selection with genetic models that consider both genetic effects may be a faster alternative for selecting clones for advancement in the breeding pipeline, parents for crossings, inheritance studies, and variation of traits at the different stages of the cassava breeding program.

Another strategy for selecting promising parents is the pedigree-based best linear unbiased prediction (P-BLUP) method for predicting breeding values (Ceballos et al., 2016a). This strategy attempts to estimate breeding values after obtaining clone phenotypic data. According to Piepho et al. (2008) this method allows for dissection of the genotypic value in additive and non-additive effects, and it can be approached by identity-by-descent (P-BLUP) or identity-by-state (G-BLUP) information. However, this method requires a large amount of kinship information, which is not always available once several crosses have been carried out between germplasm accessions with no kinship data available. Nevertheless, the lack of kinship/pedigree information can be efficiently compensated for by identity-by-state (IBS) performed using an additive relationship matrix proposed by VanRaden (2007), as Hayes et al. (2009) considered the additive genomic relationship matrix as accurate as the kinship matrix. Bouvet et al. (2016) found that the G-BLUP prediction method had higher predictive ability than P-BLUP in several genetic models. According to Zhang et al. (2015) the GEBVs may be even more accurate when using a genetic architecture-enhanced relationship matrix for each trait, with the parametrization of relationship matrix composed by markers with high effect for the trait.

Using G-BLUP for breeding value estimation at preliminary, advanced and uniform yield trials, we assume that there is a genetic correlation between clones due to relationship-by-state (Piepho et al., 2008). Since cassava is vegetatively propagated, the additive genomic matrix may be used as a genetic covariance matrix for selecting promising parents by applying mixed models in the different breeding phases (such as the clonal evaluation trial, preliminary yield trial, advanced yield trial, and uniform yield trial). As the correlation between breeding values vs. genotypic values is not perfect (0.716 of selection coincidence at 13.3% selection proportion for FRY, and 0.690 of selection coincidence at 8.3% selection proportion for DRY), the coincidence in the selection of clones to be used as parents and for advancement in the breeding program tends to be low. Therefore, by using the genetic covariance matrix in mixed models, the selection of parents with high potential to generate promising clones would be performed based on their breeding values even if the clones had low genotypic value and/or low *per se* performance. This strategy can increase the parent selection accuracy, estimate the narrow-sense heritability, and predict the GEBVs and GEGVs across the field trials, assisting parent and clone selection, respectively.

## 5 Conclusions

The genetic variances for FRY and DRY were largely derived from dominance deviations, while DMC was predominantly additive. Identification of the best genetic model allows breeders to achieve higher genetic gains in the cassava breeding program. Genomic selection can be used to assist in breeding value prediction and the selection of outstanding parents at early breeding steps, as well as to identify and select the genotypic value of good clones for advancement in the breeding pipeline. Genomic selection may achieve higher genetic gains by reducing the breeding cycle time by at least 40%.

## Data availability statement

The datasets presented in this study can be found in online repositories. The names of the repository/repositories and accession number(s) can be found below: https://figshare.com/ , doi.org/10.6084/m9.figshare.21330972.

## Author contributions

Study conception and design: LA, MR, EO. Data collection: LA, MS, EO. Analysis and interpretation of results: LA, MW, J-LJ, MR, EO. Draft manuscript preparation: LA, MS, MW. Final manuscript revision: MS, MW, CA, MR, EO. All authors reviewed the results and approved the final version of the manuscript. All authors contributed to the article and approved the submitted version.

## Supplementary material

Supplementary Figure 1 Variance components and standard deviation of genomic effects predicted by the genetic models of different genomic prediction method for fresh (FRY) and dry root yield (DRY), dry matter content (DMC) in cassava. Bayes B A: Bayes B method with additive genetic model; Bayes B A+D: Bayes B method with additive-dominant genetic model; G-BLUP A: G-BLUP method with additive genetic model; G-BLUP A+D Cla: G-BLUP method with additive-dominant classical genetic model; G-BLUP A+D Gen: G-BLUP method with additive-dominant genotypic genetic model; RKHS: reproducing kernel Hilbert spaces..

Supplementary Figure 2 Genetic gains by reducing the cassava breeding cycle takes into account the genomic (GB) and phenotypic breeding (PB) using the genomic prediction method and genetic models with higher predictive ability. G-BLUP A+D classical method for fresh root yield (FRY) and dry root yield (DRY) and G-BLUP A for dry matter content (DMC).
