# Supplementary figures and images for: Increasing cassava root yield: Additive-dominant genetic models for selection of parents and clones
Source: Front Plant Sci. 2022 Dec 16;13:1071156. doi: 10.3389/fpls.2022.1071156 (PMC9800927; doi:10.3389/fpls.2022.1071156)

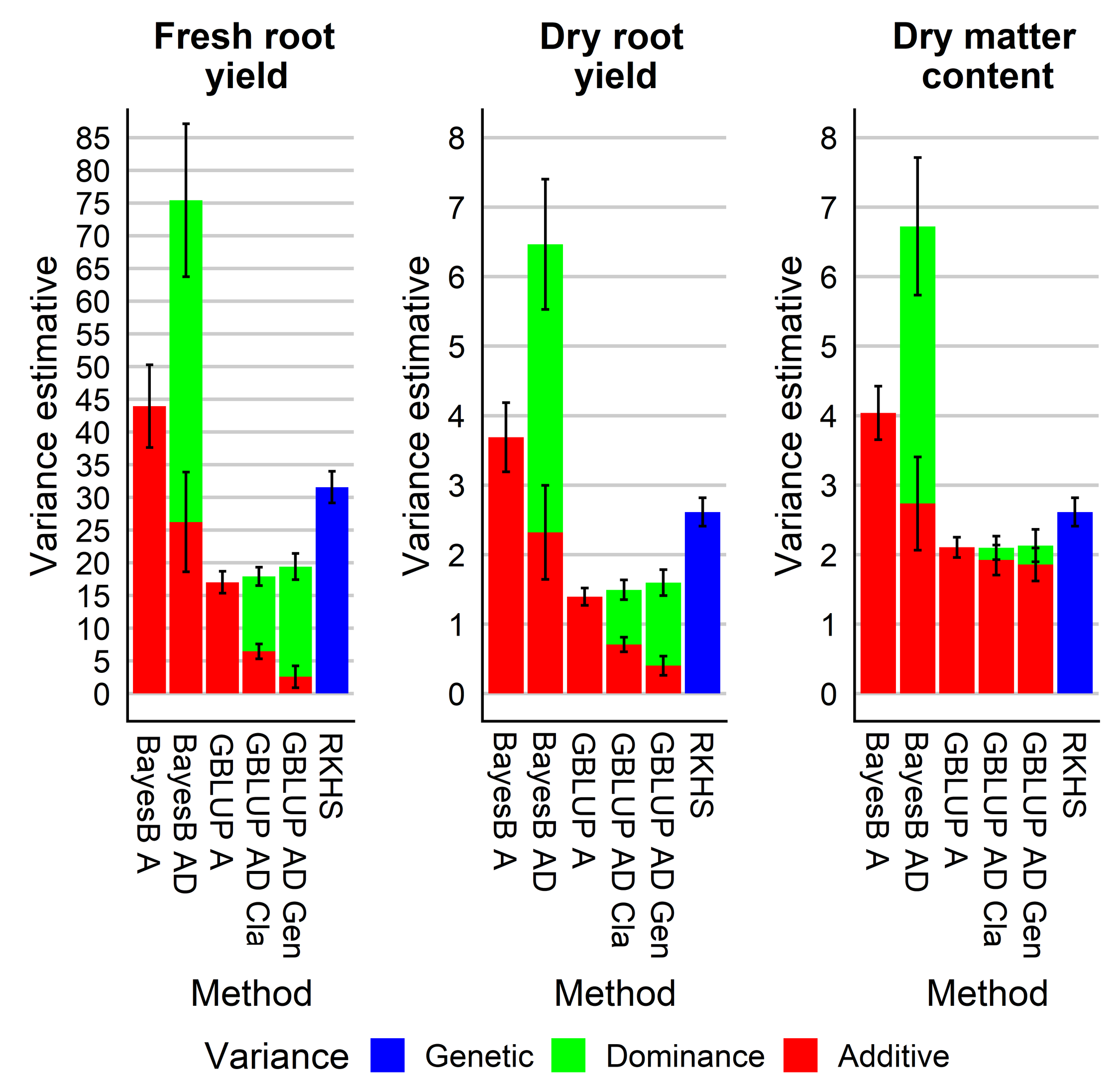

Supplement: Supplementary Figure 1 — Variance components and standard deviation of genomic effects predicted by the genetic models of different genomic prediction method for fresh (FRY) and dry root yield (DRY), dry matter content (DMC) in cassava. Bayes B A: Bayes B method with additive genetic model; Bayes B A+D: Bayes B method with additive-dominant genetic model; G-BLUP A: G-BLUP method with additive genetic model; G-BLUP A+D Cla: G-BLUP method with additive-dominant classical genetic model; G-BLUP A+D Gen: G-BLUP method with additive-dominant genotypic genetic model; RKHS: reproducing kernel Hilbert spaces.. [file Image_1.tif]

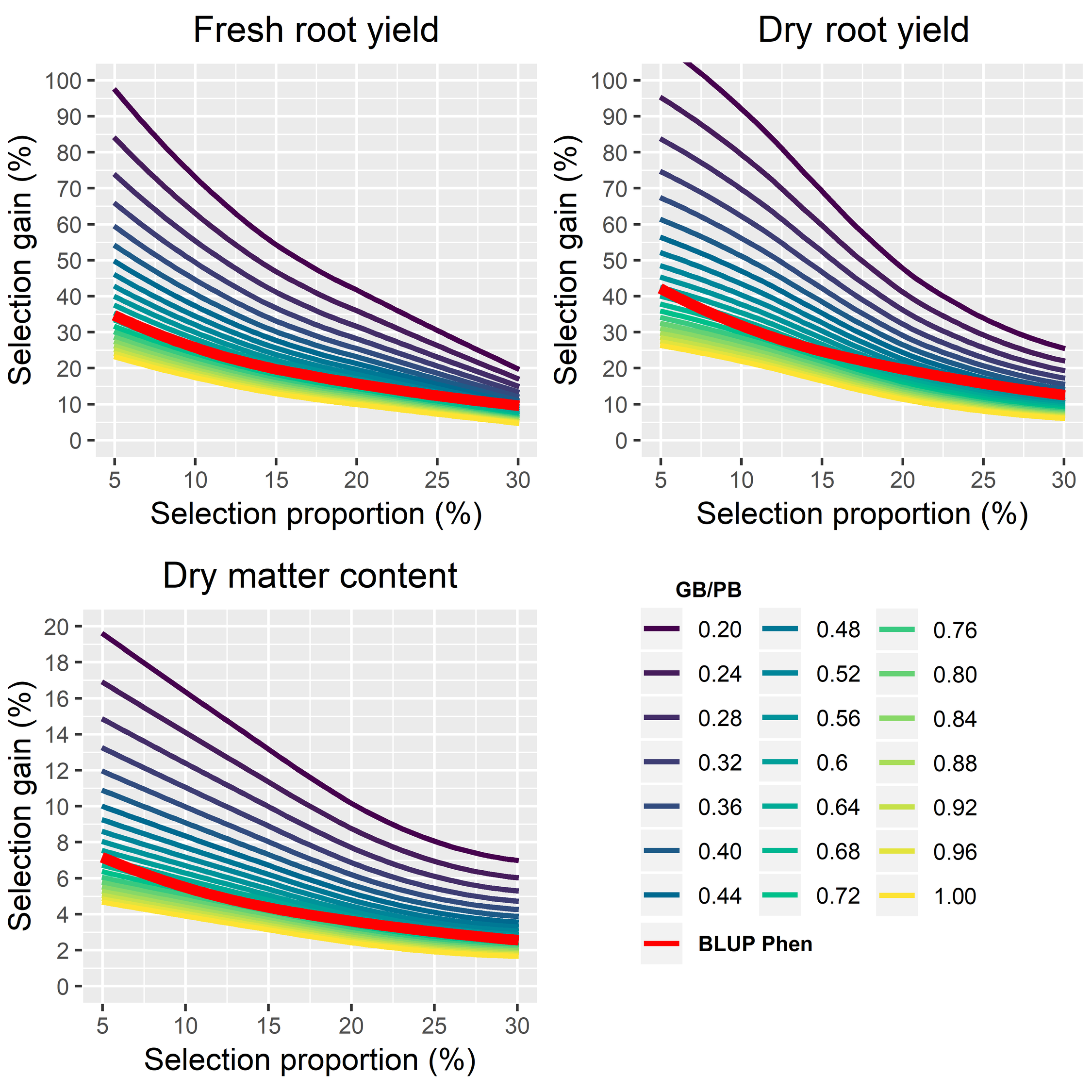

Supplement: Supplementary Figure 2 — Genetic gains by reducing the cassava breeding cycle takes into account the genomic (GB) and phenotypic breeding (PB) using the genomic prediction method and genetic models with higher predictive ability. G-BLUP A+D classical method for fresh root yield (FRY) and dry root yield (DRY) and G-BLUP A for dry matter content (DMC). [file Image_2.tif]
