# Supplementary material for: Increasing cassava root yield: Additive-dominant genetic models for selection of parents and clones
Source: Front Plant Sci. 2022 Dec 16;13:1071156. doi: 10.3389/fpls.2022.1071156 (PMC9800927; doi:10.3389/fpls.2022.1071156)
Supplement: Supplementary file 3 [file Table_1.docx]

**Supplementary material**

**Table S1.** Descriptive Analysis from 21 field trials evaluated by Embrapa Cassava between 2011 and 2016 for Fresh Root Yield (FRY), Dry Matter Content (DMC), and Dry Root Yield (DRY).

| **Year** | **Location** | **Trial** | **Design** | **Blocks** | **Clones*** | **FRY** | | **DMC** | | | **DRY** | | |
| --- | --- | --- | --- | --- | --- | --- | --- | --- | --- | --- | --- | --- | --- |
|  |  |  |  |  |  | **Mean** | **Sd** | **Mean** | **Sd** | **Mean** | | **Sd** |  |
| 2011 | Cruz das Almas | Agroverde1-CNPMF | RCBD | 3 | 233-180 | 31.9 | 18.3 | 30.7 | 3.2 | 8.5 | | 4.7 |  |
|  |  | Agroverde2-CNPMF | RCBD | 3 | 175-55 | 18.0 | 12.5 | 40.6 | 4.1 | 7.3 | | 4.5 |  |
| 2012 | Cruz das Almas | Agroverde1-CNPMF | ARCBD | 9 | 273-207 | 32.9 | 16.9 | 39.6 | 3.6 | 11.6 | | 6.0 |  |
|  |  | Agroverde2-CNPMF | ARCBD | 6 | 122-59 | 24.6 | 13.5 | 40.8 | 5.8 | 9.3 | | 5.2 |  |
|  |  | AreaCitros-CNPMF | ARCBD | 7 | 218-157 | 35.7 | 19.5 | 35.1 | 3.4 | 11.0 | | 6.3 |  |
| 2014 | Cruz das Almas | BAG1-CNPMF1 | ARCBD | 6 | 483-416 | 24.8 | 15.2 | 35.1 | 3.2 | 7.6 | | 4.8 |  |
|  |  | BAG1-CNPMF2 | ARCBD | 10 | 513-426 | 20.1 | 10.2 | 34.7 | 3.3 | 6.1 | | 3.1 |  |
|  |  | BAG2-UFRB | ARCBD | 10 | 438-240 | 21.3 | 8.8 | 35.4 | 2.7 | 6.6 | | 2.9 |  |
|  | Laje | BAG1-Coopamido | ARCBD | 10 | 589-525 | 14.9 | 7.6 | 36.1 | 2.7 | 4.7 | | 2.5 |  |
|  |  | BAG2-Coopamido | ARCBD | 8 | 188-181 | 20.6 | 9.6 | 36.3 | 3.0 | 6.7 | | 3.3 |  |
|  |  | BAG3-Coopamido | RCBD | 2 | 356-339 | 35.1 | 16.6 | 32.9 | 3.3 | 10.0 | | 5.1 |  |
| 2015 | Cruz das Almas | BAG1-1 | ARCBD | 10 | 598-475 | 16.6 | 9.2 | 34.4 | 2.9 | 5.0 | | 2.8 |  |
|  |  | BAG1-2 | ARCBD | 10 | 497-417 | 15.5 | 8.5 | 35.4 | 2.7 | 4.8 | | 2.8 |  |
|  |  | BAG2-UFRB | ARCBD | 10 | 431-234 | 21.1 | 10.1 | 36.1 | 2.4 | 6.7 | | 3.4 |  |
|  |  | BAG4-CNPMF | ARCBD | 13 | 723-621 | 19.5 | 13.8 | 32.3 | 3.1 | 5.5 | | 4.0 |  |
|  | Laje | BAG1-NovoHorizonte | ARCBD | 12 | 556-504 | 21.8 | 11.1 | 35.9 | 2.7 | 6.9 | | 3.7 |  |
|  |  | BAG2-NovoRumo | ARCBD | 6 | 179-174 | 23.4 | 10.1 | 33.9 | 2.9 | 6.8 | | 3.0 |  |
|  |  | BAG3-NovoHorizonte | ARCBD | 5 | 369-350 | 22.6 | 11.3 | 35.0 | 3.1 | 6.9 | | 3.6 |  |
| 2016 | Cruz das Almas | BAG1-1 | ARCBD | 9 | 486-424 | 7.8 | 6.3 | 31.5 | 2.5 | 2.1 | | 1.7 |  |
|  |  | BAG1-2 | ARCBD | 10 | 550-424 | 15.5 | 8.8 | 34.1 | 2.6 | 4.6 | | 2.6 |  |
|  |  | BAG-4 | ARCBD | 12 | 652-564 | 7.4 | 5.8 | 34.4 | 3.3 | 2.4 | | 1.8 |  |

RCBD: Randomized Complete Block Design; ARCBD: Augmented Randomized Complete Block Design; *First number is related to the total clone number evaluated on the respective trial, while the second number is the total number of clones from the genomic selection Training population (888 clones in total) present at the respective trial.
