# Supplementary material for: Increasing cassava root yield: Additive-dominant genetic models for selection of parents and clones
Source: Front Plant Sci. 2022 Dec 16;13:1071156. doi: 10.3389/fpls.2022.1071156 (PMC9800927; doi:10.3389/fpls.2022.1071156)
Supplement: Supplementary file 4 [file Table_2.docx]

**Supplementary material**

**Table S2.** Coincidence of Clones between the 21 field trials evaluated by Embrapa Cassava between 2011 and 2016 for Fresh Root Yield (FRY), Dry Matter Content (DMC), and Dry Root Yield (DRY).

| **N** | **Year** |  |  | **2011** | | **2012** | | | **2014** | | | | | |
| --- | --- | --- | --- | --- | --- | --- | --- | --- | --- | --- | --- | --- | --- | --- |
|  |  | **Location** |  | **Cruz das Almas** | | **Cruz das Almas** | | | **Cruz das Almas** | | | **Laje** | | |
|  |  |  | **Trial** | **1** | **2** | **3** | **4** | **5** | **6** | **7** | **8** | **9** | **10** | **11** |
| 1 | 2011 | Cruz das Almas | Agroverde1-CNPMF | 180-233 | 6 | 202 | 4 | 16 | 32 | 32 | 195 | 44 | 147 | 33 |
| 2 |  |  | Agroverde2-CNPMF | 6 | 55-175 | 6 | 79 | 3 | 16 | 81 | 15 | 10 | 6 | 6 |
| 3 | 2012 | Cruz das Almas | Agroverde1-CNPMF | 159 | 5 | 207-273 | 10 | 17 | 40 | 54 | 234 | 64 | 162 | 48 |
| 4 |  |  | Agroverde2-CNPMF | 4 | 39 | 10 | 59-122 | 6 | 16 | 65 | 16 | 12 | 8 | 14 |
| 5 |  |  | AreaCitros-CNPMF | 16 | 3 | 17 | 6 | 157-218 | 27 | 159 | 21 | 16 | 16 | 16 |
| 6 | 2014 | Cruz das Almas | BAG1-CNPMF1 | 32 | 15 | 40 | 14 | 27 | 416-483 | 131 | 62 | 391 | 39 | 219 |
| 7 |  |  | BAG1-CNPMF2 | 32 | 50 | 54 | 55 | 149 | 131 | 426-513 | 76 | 222 | 47 | 144 |
| 8 |  |  | BAG2-UFRB | 163 | 10 | 190 | 14 | 21 | 61 | 74 | 240-438 | 90 | 176 | 62 |
| 9 |  | Laje | BAG1-Coopamido | 44 | 10 | 63 | 12 | 16 | 374 | 214 | 85 | 525-589 | 50 | 305 |
| 10 |  |  | BAG2-Coopamido | 143 | 6 | 159 | 8 | 16 | 39 | 47 | 175 | 50 | 181-188 | 36 |
| 11 |  |  | BAG3-Coopamido | 33 | 6 | 48 | 14 | 16 | 218 | 140 | 61 | 302 | 36 | 339-356 |
| 12 | 2015 | Cruz das Almas | BAG1-1 | 38 | 22 | 48 | 19 | 31 | 415 | 165 | 71 | 415 | 46 | 254 |
| 13 |  |  | BAG1-2 | 33 | 49 | 53 | 55 | 149 | 126 | 410 | 72 | 206 | 46 | 135 |
| 14 |  |  | BAG2-UFRB | 164 | 10 | 190 | 11 | 19 | 59 | 67 | 233 | 82 | 172 | 58 |
| 15 |  |  | BAG4-CNPMF | 66 | 10 | 91 | 16 | 93 | 370 | 291 | 107 | 488 | 69 | 313 |
| 16 |  | Laje | BAG1-NovoHorizonte | 41 | 9 | 63 | 14 | 16 | 365 | 202 | 83 | 499 | 50 | 293 |
| 17 |  |  | BAG2-NovoRumo | 132 | 6 | 149 | 8 | 16 | 42 | 48 | 165 | 52 | 164 | 35 |
| 18 |  |  | BAG3-NovoHorizonte | 33 | 6 | 50 | 14 | 17 | 227 | 146 | 66 | 313 | 38 | 336 |
| 19 | 2016 | Cruz das Almas | BAG1-1 | 32 | 22 | 39 | 19 | 26 | 376 | 122 | 62 | 374 | 38 | 222 |
| 20 |  |  | BAG1-2 | 37 | 49 | 57 | 55 | 147 | 133 | 400 | 76 | 209 | 49 | 138 |
| 21 |  |  | BAG-4 | 60 | 9 | 84 | 16 | 84 | 331 | 265 | 98 | 445 | 66 | 289 |

Upper diagonal elements are the total clone coincidence between trials, while lower diagonal elements are the clone coincidence limited to the training population selected for the Genomic Prediction analysis.

**Table S2.** Coincidence of Clones between the 21 field trials evaluated by Embrapa Cassava between 2011 and 2016 for Fresh Root Yield (FRY), Dry Matter Content (DMC), and Dry Root Yield (DRY). **Cont...**

| **N** | **Year** |  |  | **2015** | | | | | | | **2016** | | | |
| --- | --- | --- | --- | --- | --- | --- | --- | --- | --- | --- | --- | --- | --- | --- |
|  |  | **Location** |  | **Cruz das Almas** | | | | **Laje** | | | **Cruz das Almas** | | | |
|  |  |  | **Trial** | **12** | **13** | **14** | **15** | **16** | **17** | **18** | **19** | **20** | **21** |  |
| 1 | 2011 | Cruz das Almas | Agroverde1-CNPMF | 38 | 33 | 193 | 70 | 41 | 135 | 33 | 32 | 38 | 66 |  |
| 2 |  |  | Agroverde2-CNPMF | 25 | 80 | 14 | 11 | 9 | 6 | 6 | 24 | 76 | 10 |  |
| 3 | 2012 | Cruz das Almas | Agroverde1-CNPMF | 49 | 53 | 230 | 95 | 63 | 151 | 50 | 39 | 58 | 88 |  |
| 4 |  |  | Agroverde2-CNPMF | 21 | 65 | 12 | 17 | 14 | 8 | 14 | 19 | 63 | 18 |  |
| 5 |  |  | AreaCitros-CNPMF | 34 | 157 | 19 | 121 | 16 | 16 | 17 | 26 | 152 | 107 |  |
| 6 | 2014 | Cruz das Almas | BAG1-CNPMF1 | 481 | 126 | 60 | 378 | 379 | 42 | 227 | 407 | 156 | 338 |  |
| 7 |  |  | BAG1-CNPMF2 | 175 | 481 | 68 | 301 | 208 | 48 | 149 | 124 | 463 | 273 |  |
| 8 |  |  | BAG2-UFRB | 83 | 72 | 410 | 110 | 88 | 166 | 67 | 64 | 80 | 101 |  |
| 9 |  | Laje | BAG1-Coopamido | 445 | 212 | 85 | 511 | 550 | 52 | 317 | 399 | 215 | 461 |  |
| 10 |  |  | BAG2-Coopamido | 46 | 47 | 173 | 70 | 50 | 169 | 38 | 38 | 50 | 70 |  |
| 11 |  |  | BAG3-Coopamido | 257 | 138 | 58 | 322 | 295 | 35 | 350 | 223 | 141 | 295 |  |
| 12 | 2015 | Cruz das Almas | BAG1-1 | 475-598 | 158 | 74 | 437 | 423 | 48 | 265 | 477 | 200 | 387 |  |
| 13 |  |  | BAG1-2 | 155 | 417-497 | 65 | 294 | 201 | 48 | 145 | 120 | 464 | 267 |  |
| 14 |  |  | BAG2-UFRB | 70 | 65 | 234-431 | 107 | 83 | 163 | 64 | 63 | 74 | 97 |  |
| 15 |  |  | BAG4-CNPMF | 416 | 285 | 104 | 621-723 | 487 | 72 | 336 | 380 | 297 | 627 |  |
| 16 |  | Laje | BAG1-NovoHorizonte | 401 | 196 | 80 | 469 | 504-556 | 52 | 308 | 380 | 200 | 441 |  |
| 17 |  |  | BAG2-NovoRumo | 48 | 47 | 162 | 71 | 52 | 174179 | 38 | 39 | 52 | 73 |  |
| 18 |  |  | BAG3-NovoHorizonte | 264 | 142 | 63 | 325 | 305 | 38 | 350-369 | 233 | 150 | 308 |  |
| 19 | 2016 | Cruz das Almas | BAG1-1 | 422 | 120 | 60 | 369 | 361 | 39 | 232 | 424-486 | 127 | 340 |  |
| 20 |  |  | BAG1-2 | 167 | 397 | 72 | 288 | 195 | 51 | 145 | 124 | 424-550 | 274 |  |
| 21 |  |  | BAG-4 | 374 | 260 | 95 | 553 | 428 | 69 | 301 | 332 | 265 | 564-652 |  |

Upper diagonal elements are the total clone coincidence between trials, while lower diagonal elements are the clone coincidence limited to the training population selected for the Genomic Prediction analysis.
