# Supplementary material for: Increasing cassava root yield: Additive-dominant genetic models for selection of parents and clones
Source: Front Plant Sci. 2022 Dec 16;13:1071156. doi: 10.3389/fpls.2022.1071156 (PMC9800927; doi:10.3389/fpls.2022.1071156)
Supplement: Supplementary file 5 [file Table_3.docx]

**Supplementary material**

**Table S3**. Prior of π assumed for Bayes B prediction for fresh root yield (FRY), dry root yield (DRY), and dry matter content (DMC) in roots of cassava.

| π prior | FRY | DRY | DMC |
| --- | --- | --- | --- |
| Additive effect | 0.4587 | 0.4524 | 0.55 |
| Dominant effect | 0.4684 | 0.4661 | 0.4563 |
