# Supplementary material for: Increasing cassava root yield: Additive-dominant genetic models for selection of parents and clones
Source: Front Plant Sci. 2022 Dec 16;13:1071156. doi: 10.3389/fpls.2022.1071156 (PMC9800927; doi:10.3389/fpls.2022.1071156)
Supplement: Supplementary file 6 [file Table_4.docx]

**Supplementary material**

**Table S4.** Expected selection gains (%) of different genomic prediction methods and genetic models for fresh root yield (FRY), dry root yield (DRY) and dry matter content (DMC) in cassava, with a selection proportion of 5-30%.

|  | Fresh root yield | | | | | |  | Dry root yield | | | | | |  | Dry matter content | | | | | |
| --- | --- | --- | --- | --- | --- | --- | --- | --- | --- | --- | --- | --- | --- | --- | --- | --- | --- | --- | --- | --- |
| SP | 5% | 10% | 15% | 20% | 25% | 30% |  | 5% | 10% | 15% | 20% | 25% | 30% |  | 5% | 10% | 15% | 20% | 25% | 30% |
| G-BLUP A | 20.3 | 14.6 | 9.6 | 6.9 | 4.5 | 2.3 |  | 22.7 | 18.3 | 11.5 | 8.3 | 6.5 | 4.6 |  | 4.6 | 3.9 | 3.1 | 2.4 | 1.9 | 1.6 |
| G-BLUP A+D Clas | 23.8 | 17.2 | 13.0 | 9.9 | 7.4 | 4.7 |  | 25.8 | 22.0 | 16.7 | 11.3 | 8.4 | 5.8 |  | 4.6 | 3.9 | 3.1 | 2.4 | 1.9 | 1.6 |
| G-BLUP A+D Gen | 22.3 | 17.2 | 12.5 | 9.0 | 7.0 | 3.8 |  | 24.3 | 21.9 | 16.5 | 10.6 | 8.1 | 5.7 |  | 4.6 | 3.9 | 3.1 | 2.4 | 1.9 | 1.6 |
| Bayes B A | 21.2 | 15.6 | 10.4 | 7.2 | 5.0 | 3.0 |  | 23.5 | 19.6 | 12.8 | 9.3 | 6.7 | 4.8 |  | 4.6 | 3.9 | 3.1 | 2.4 | 1.9 | 1.6 |
| Bayes B A+D | 23.8 | 18.4 | 13.3 | 10.4 | 7.8 | 5.3 |  | 26.2 | 23.0 | 17.7 | 13.0 | 8.8 | 6.5 |  | 4.7 | 4.0 | 3.3 | 2.6 | 2.2 | 1.8 |
| RKHS | 22.7 | 17.2 | 12.5 | 9.1 | 6.5 | 4.5 |  | 25.0 | 22.5 | 16.5 | 10.6 | 8.1 | 5.5 |  | 4.6 | 4.0 | 3.3 | 2.6 | 2.1 | 1.7 |
| BLUP Phen | 35.1 | 25.4 | 19.8 | 15.7 | 12.4 | 9.6 |  | 42.8 | 31.7 | 24.7 | 19.7 | 15.8 | 12.5 |  | 7.3 | 5.5 | 4.4 | 3.6 | 3.0 | 2.5 |

SP: selection proportion; G-BLUP A: G-BLUP method with additive genetic model; G-BLUP A+D Clas: G-BLUP method with additive-dominant classical genetic model; G-BLUP A+D Gen: G-BLUP with additive- dominant genotypic genetic model; Bayes B A: Bayes B method with additive genetic model; Bayes B A+D: Bayes B method with additive-dominant genetic model; RKHS: reproducing kernel Hilbert spaces; BLUP Phen: phenotypic BLUP estimated by mixed models.
